# Effects of the *Staphylococcus aureus* and *Staphylococcus epidermidis* Secretomes Isolated from the Skin Microbiota of Atopic Children on CD4^+^ T Cell Activation

**DOI:** 10.1371/journal.pone.0141067

**Published:** 2015-10-28

**Authors:** Emeline Laborel-Préneron, Pascale Bianchi, Franck Boralevi, Philippe Lehours, Frédérique Fraysse, Fanny Morice-Picard, Motoyuki Sugai, Yusuke Sato'o, Cédric Badiou, Gérard Lina, Anne-Marie Schmitt, Daniel Redoulès, Christiane Casas, Christian Davrinche

**Affiliations:** 1 INSERM UMR 1043, CNRS UMR 5282, Université Toulouse III Paul Sabatier, Toulouse, France; 2 Dermo-Cosmétique, Pierre Fabre, Toulouse, France; 3 Pediatric Dermatology unit, Pellegrin Children's Hospital, Bordeaux, France; 4 Bacteriology department, CHU Bordeaux, Bordeaux, France; 5 Department of Bacteriology, Hiroshima University Graduate School of Biomedical & Health Sciences, Kasumi 1-2-3, Minami-ku, Hiroshima City, Hiroshima, Japan 734–8551; 6 INSERM U1111, Université Lyon 1, Hospices Civils de Lyon, France; Virginia Tech University, UNITED STATES

## Abstract

Interactions between the immune system and skin bacteria are of major importance in the pathophysiology of atopic dermatitis (AD), yet our understanding of them is limited. From a cohort of very young AD children (1 to 3 years old), sensitized to *Dermatophagoides pteronyssinus* allergens (Der p), we conducted culturomic analysis of skin microbiota, cutaneous transcript profiling and quantification of anti-Der p CD4^+^ T cells. This showed that the presence of *S*. *aureus* in inflamed skin of AD patients was associated with a high IgE response, increased expression of inflammatory and Th2/Th22 transcripts and the prevalence of a peripheral Th2 anti-Der p response. Monocyte-derived dendritic cells (moDC) exposed to the *S*. *aureus* and *S*. *epidermidis* secretomes were found to release pro-inflammatory IFN-γ and anti-inflammatory IL-10, respectively. Allogeneic moDC exposed to the *S*. *aureus* secretome also induced the proliferation of CD4^+^ T cells and this effect was counteracted by concurrent exposure to the *S*. *epidermidis* secretome. In addition, whereas the *S*. *epidermidis* secretome promoted the activity of regulatory T cells (Treg) in suppressing the proliferation of conventional CD4^+^ T cells, the Treg lost this ability in the presence of the *S*. *aureus* secretome. We therefore conclude that *S*. *aureus* may cause and promote inflammation in the skin of AD children through concomitant Th2 activation and the silencing of resident Treg cells. Commensals such as *S*. *epidermidis* may counteract these effects by inducing the release of IL-10 by skin dendritic cells.

## Introduction

Atopic dermatitis (AD) is an inflammatory skin disorder whose prevalence has increased in the last few decades and today affects 10–30% of children in developed countries. The complexity of AD pathogenesis has been extensively highlighted and results from a combination of genetic and acquired defects including barrier dysfunction, inappropriate and inefficient innate response to allergens and pathogens, and activation of Th2 lymphocytes with inhibitory effect on the production of antimicrobial peptides by keratinocytes. Together these defects create and sustain a vicious cycle that contributes to the severity of the disease [1–4]. Recent studies have shown that the skin of healthy adults contains a billion long-term persisting T cells and have clearly demonstrated a role for them in both normal immunity and inflammatory skin diseases [5–7]. Indeed, resident effector memory T cells directed against antigens derived from skin pathogens can provide immediate protection against viral and bacterial infections. Regulatory T cells (Treg) are also present and control the unusual activation and expansion of auto-reactive and inflammatory T cells [8, 9]. In the pathophysiology of AD, the expansion and activation of resident Th2 lymphocytes are key effectors in the progression of the disease. Therefore, the secretion of IL-10, which inhibits Th2 proliferation, by cells such as Treg and antigen presenting cells (APC) is essential for suppressing skin inflammation to prevent AD. However, recent findings have revealed that bacteria from the normal skin microbiota such as *Staphylococcus epidermidis* (*S*. *epidermidis*) could help to suppress the inflammatory process through a mutualistic role with the host to inducing the secretion of anti-inflammatory IL-10 by APCs [10, 11]. This anti-inflammatory activity of *S*. *epidermidis* and the education of the host immune system are, at least in part, mediated through secreted factors such as peptidoglycan, the lipopeptide LP01 and lipoteichoic acid (LTA). Moreover, these commensals are also thought to control the growth of *Staphylococcus aureus (S*. *aureus)*, a skin pathogen that is highly prevalent in AD patients [12]. The characterization of skin bacterial communities associated with AD has demonstrated that *S*. *aureus* is dominant during flares, in contrast to the bacterial diversity found in control and post-flare subjects [13]. *S*. *aureus* secretes virulence factors which promote AD, such as α-toxin and protein A (Spa), that are involved in immune evasion, and δ-toxin which can exacerbate the allergic response. Superantigens produced by *S*. *aureus* also play an important role in the T cell response. These findings have provided new insights on how the composition and diversity of the skin microbiota and its interplay with skin-associated lymphoid cells could modulate the inflammatory immune response in AD.

In this study we examined the skin microbiota in very young AD children (from 1 to 3 years old) with respect to cutaneous inflammatory transcripts and peripheral CD4^+^ T cell responses to skin allergens. We studied CD4^+^ T cell-specific responses by ELISpot assay, using allergens of the house dust mite *Dermatophagoides pteronyssinus* since these are major aeroallergens in AD pathophysiology. *In vitro* experiments using *S*. *aureus* and *S*. *epidermidis* secretomes isolated from patients revealed that the deleterious effects of *S*. *aureus* on monocyte-derived dendritic cell (moDC) function could be counteracted by *S*. *epidermidis*-induced IL-10 secretion. Therefore, promoting the anti-inflammatory properties of cutaneous commensals such as *S*. *epidermidis* could prevent inflammatory progression in the skin of atopic patients.

## Materials and Methods

### Study participants and sample collection

This study was carried out from March 2012 to June 2013 in the Pediatric Dermatology Unit of the Children’s Hospital in Bordeaux (France). The investigators were experienced dermatologists who recruited children from the patients they usually followed-up on. This study was conducted according to the principles of the Declaration of Helsinki and its subsequent amendments and the guidelines for Good Clinical Practices (CPMP/ICH/135/95), and was in line with French regulations. The protocol was approved by the South West Ethics Committee and Overseas III in Bordeaux, France (N°CPP 2011/102) and by the French Agency for the Safety of Health Products (AFSSAPS) (Ref B111515-30). Patients were included after their parents or guardians had signed a written informed consent form. Subjects included in this study involved a group of 21 children (subject 33 was not included), aged from 12 to 36 months old (mean age = 24.1 months), suffering from AD according to the UK Working Party criteria with a SCORAD index (which indicates the severity of AD) between 20 and 40. Children who were sensitized to *D*. *pteronyssinus* were chosen as defined as defined by a positive skin prick test response (wheal diameter > 3mm), a positive atopy patch test or a previous positive RAST. A second group of 17 age-matched healthy children (mean age = 24.94 months) with no personal or familial history of AD in first-degree relatives, no food allergies other than cow’s milk allergy, no asthma, and no skin disease related to defects in skin barrier function, an abnormal skin microbiota and/or an inflammatory syndrome. None of the children involved in the study had received application of topical corticosteroid, antiseptic, immunosuppressive, antifungal or antibiotic treatments within the 7 days prior to inclusion. Systemic immunosuppressive, immunomodulator, antifungal and antibiotic treatments were also stopped 15 days prior to inclusion. Children were also not included if any skin care product or topical treatment had been applied after the last toilet the day before the inclusion visit except on the nappy area. Skin and blood samples were collected at time of inclusion and IgE quantification was carried out by the Immunology Laboratory of Bordeaux CHU Pellegrin. For AD subjects, skin samples were taken from AD skin lesions that were “active” lesions, namely erythematous and pruriginous lesions, with scales and/or oozing, with no consideration of the delay of onset. Most of the sampled lesions were acute lesions, but persisting lesions with recent flares may have been sampled in some cases. Samples from healthy children were from matched zones. Blood samples from subjects 1, 3 and 7 could not be used in ELISpot assays due to transportation issues. Total IgE quantification could not be obtained for subjects 3 and 16. The bacterial composition of skin was determined from swab sampling, a technique which had previously been successfully used for the identification of skin bacterial populations [14].

### Culturomics of skin bacterial isolates

Skin samples were recovered using cotton swabs from the inflamed areas of AD children and matched areas on non-AD controls. Swabs were either maintained at 4°C or frozen at -20°C before seeding. After Gram staining, swabs were quantitatively seeded on non-selective media under aerobic or anaerobic conditions (Trypticase Soy Agar with 5% horse blood (BioMérieux, Marcy L’Etoile, France) and Schaedler with vitamin K1 and 5% Sheep Blood (Becton Dickinson, Le Pont de Claix, France) agar plates) for 2 to 5 days, respectively. Bacterial colonies were identified on agar plates and the number of colonies grown was expressed in CFU/ml. MALDI-TOF-MS identification was carried out on a Microflex mass spectrometer (Bruker Daltonics, Bremen, Germany) using freshly isolated colonies. Briefly, a colony was added to a matrix and applied to a metal plate before ionization with a laser so that the total protein content could be analyzed by measuring their time of flight. Identification of clinical isolates was achieved by comparing the mass spectra obtained to reference spectra in the database, using MALDI Biotyper 2.0 (Bruker Daltonics). The degree of spectral concordance was expressed as a logarithmic identification score (LogScore) according to the manufacturer’s instructions.

### Real-time RT-PCR analysis of skin scratches

Skin samples were taken by scratching the skin surface (5 times back and forth) with a sterile micro-abrasive tool (Vitry, Paris, France). PCR was performed on reverse-transcribed total RNA as described in the Supporting Information files (S1 and S2 Files).

### Preparation and characterization of the bacterial secretome

Secretome samples were prepared from *S*. *aureus* and *S*. *epidermidis* clones isolated from AD and non-AD children by overnight culture until stationary phase was reached in RPMI1640 medium supplemented with 10% FCS. The quantification of enterotoxins in the *S*. *aureus* secretome, the expression of recombinant enterotoxins, and the preparation of antibodies for ELISA and Sandwich ELISA, were carried out as described in Supporting Information files (S1 and S2 Files).

### DNA microarray assay

Bacterial DNA was extracted according to the manufacturer’s protocol. Diagnostic DNA microarrays using a *S*. *aureus* Genotyping kit (Identibac *S*. *aureus* Genotyping ®, Alere), was used for superantigen gene detection as detailed in Supporting information files (S1 and S2 Files)

### Production of recombinant enterotoxins

SEB, SEC, SEG, SEI, SElM, SElN and SElO were produced in *Escherichia coli* M15 as His-tagged recombinant toxins and purified by affinity chromatography as detailed in Supporting information files (S1 and S2 Files).

### Monocytes and monocyte-derived dendritic cells (moDC)

Monocytes and moDC were prepared from peripheral blood mononuclear cells (PBMC) and stimulated with the prepared secretomes as described in the Supporting Information file (S1 File).

### CD4^+^ T cells

Total CD4^+^ T cells or naïve CD4^+^CD45RA^+^ T cells were isolated from PBMC with the use of naive CD4^+^ T cell isolation kit II and CD4 T cell isolation kit II (Miltenyi). For proliferation assays CD4^+^ T cells were stained with CFSE (Invitrogen) and co-cultured with either allogeneic moDC or monocytes for 5 days in 96-well plates at a ratio of 1 stimulator to 10 T cells. Treg cells were isolated with the use of a CD4^+^CD25^+^CD127^dim/neg^ Regulatory T Cell Isolation Kit II (Miltenyi) and magnetic sorting. Depending on the sorting rate, Treg cells were expanded in complete RPMI supplemented with 500 U/ml of rIL-2 and a Miltenyi Treg expansion kit. More than 95% of purified cells were FoxP3^pos^. Cells were stimulated with the *S*. *aureus* or *S*. *epidermidis* secretome (5% v/v) for 24 hours, then washed and co-cultured for 5 days with allogeneic conventional CD4^+^ T cells stained with CFSE and beads pre-loaded with anti-CD2, -CD3 and -CD28 antibodies (Treg suppression inspector, Miltenyi) at different ratios. CFSE proliferation was assessed by flow cytometry.

### Flow cytometry analysis

Cells were stained with monoclonal antibodies directed against the human antigens: CD1a, HLA-DR, CD86, CD14, CD83, CD4, CD127, CD25 and Foxp3, as detailed in Table 1. For Foxp3 staining, the Human Foxp3 buffer set (BD) was used. Analyses were performed with FACSCalibur or LSRII flow cytometers (BD Biosciences).

**Table 1:** List of antibodies used.

Antibody reactivity	Fluorochrome	Host species	Supplier	Catalogue number	Dilution
CD1a	PE	mouse	Biolegend	300106	1/100
HLA-DR (HLA-DRα)	FITC	mouse	Biolegend	307604	1/50
CD86	APC	mouse	Biolegend	305412	1/100
CD14	FITC	mouse	Biolegend	301803	1/100
CD83	PE	mouse	eBioscience	12–0839	3/50
CD4	Alexa Fluor 488	mouse	BD Pharmingen	557695	1/25
CD127 (IL-7Rα)	PE	mouse	BD Pharmingen	557938	1/50
CD25 (IL-2Rα)	APC	mouse	BD Pharmingen	555434	1/25
Foxp3	PE-CF594	mouse	BD Horizon	562421	1/25

### T cell ELISpot assays

Monocytes and autologous CD4^+^ T cells were isolated from patient/control blood within 24 hours of sampling and ELISpot assays were carried out against crude extracts of *D*. *pteronyssinus*, as described in the Supporting Information file (S1 File).

### Cytokine quantification

The production of cytokines (IFN-γ, TNF-α, IL-6, IL-12, IL-10 and IL-4) was quantified with cytometric bead array kits (CBA, BD Biosciences) and ELISA (Gen-Probe Diaclone) as indicated.

### Statistical Analysis

Statistical analyses were performed using SAS software, release 9.3, or Prism software as detailed in S1 File. All statistical tests were performed using a significance level of p<0.05.

## Results

### Skin microbiota and inflammatory profiles and peripheral allergen-specific CD4^+^ T cell responses in AD children

**Fig 1:**
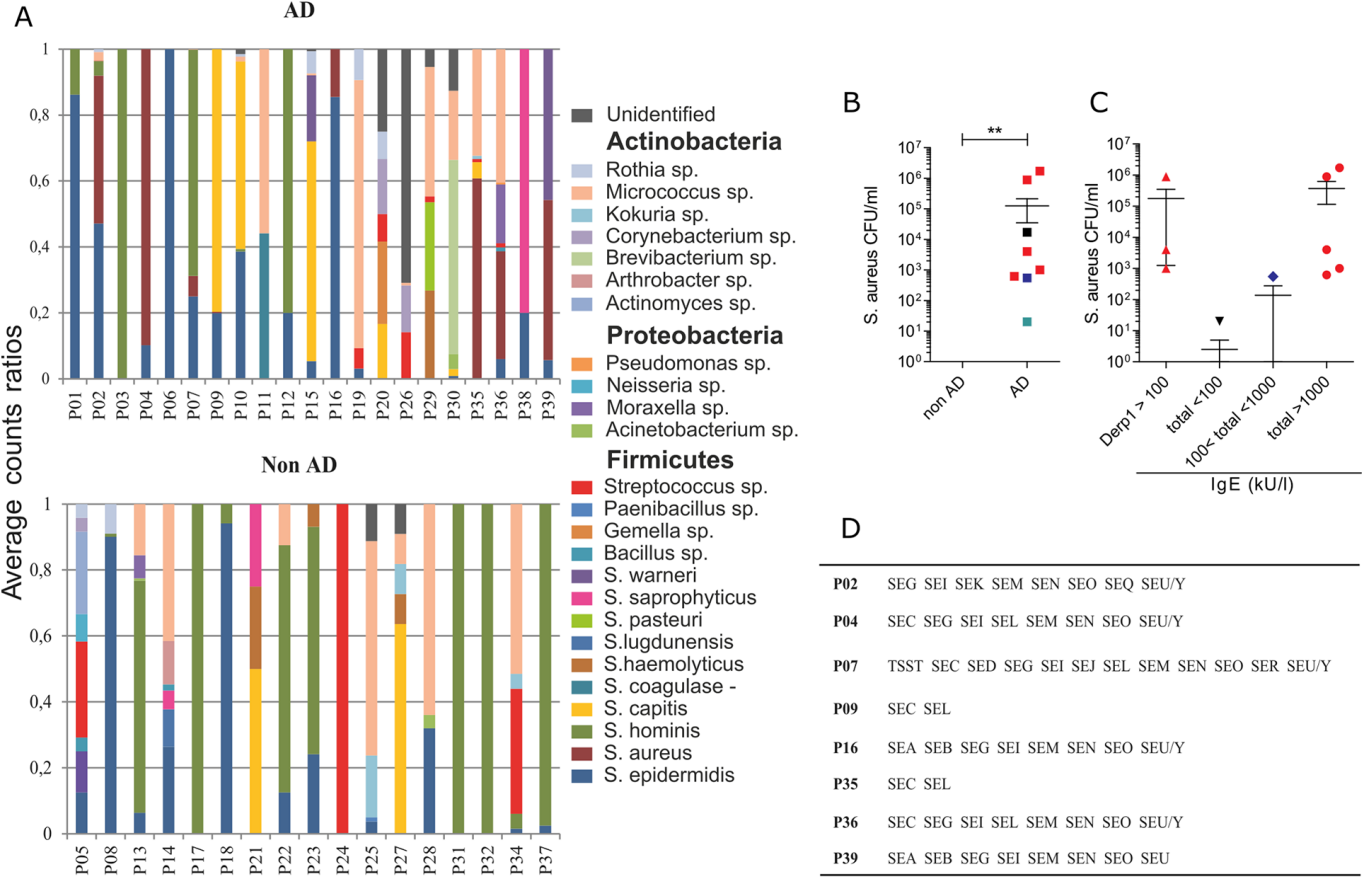
*S*. *aureus* was identified only in AD children and correlated with high IgE levels. (A) Ratios of average counts for bacterial species and their respective phyla found in the inflamed zones and control-matched zones of atopic dermatitis (AD) patients and their non-AD counterparts, respectively. (B) Colony-forming units (CFU/ml) of *S*. *aureus* from sampled zones in AD and non-AD subjects. Mann-Whitney, mean values with SEM are shown, p**<0.01. The black square denotes a patient with undetermined IgE values. (C) AD subjects were sorted in relation to the amounts of IgE antibodies (kU/l) measured in their blood. (B, C) Total IgE over 1000kU/l is shown in red, between 100 and 1000 kU/l in blue and below 100 kU/l in green. (D) Enterotoxins were identified in *S*. *aureus* clones from AD subjects (P02-P39) by DNA array.

Although AD most commonly affects very young children, few reports are available that provide an overall picture of their cutaneous microbiota, inflammatory and immune profiles. In a cohort of very young AD children (N = 21, mean age = 24.1 months, mean SCORAD score = 26) sensitized to allergens of the house dust mite *D*. *pteronyssinus* (Der p, Table 2), and their non-AD counterparts (N = 17, mean age = 24.9 months), we performed a culturomic analysis of the bacterial composition of inflamed AD patient skin and matched zones from controls. Among the 25 species found to belong to the three major phyla, *Actinobacteria*, *Firmicutes* and *Proteobacteria* (Fig 1A), *S*. *aureus* was identified only on the skin of AD patients, whereas *S*. *epidermidis* was the only species that was persistently observed in both AD and non-AD children when looking at several skin zones including xerotic and non-inflammatory ones (not shown). *S*. *aureus* was identified in 38% of AD patients (Wilcoxon, p = 0.010) but not in the control-matched zones of healthy subjects (Fig 1B). Interestingly, the highest *S*. *aureus* counts were associated with the highest total IgE levels (Spearman’s correlation, r = 0.67, p = 0.05) (Fig 1C). However, the exact Der p1-specific IgE levels were not obtained over 100kU/l, therefore we cannot claim that *S*. *aureus* counts were associated with Der p1-specific IgE rather than total IgE levels. Analysis of *S*. *aureus* clones obtained from eight identified carriers revealed the presence of superantigens including those encoded by the enterotoxin gene cluster (*egc*: SEG, SEI, SEM, SEN and SEO, Fig 1D) [15]. This observation confirms the frequent association of superantigen producing strains with AD [16].

**Table 2:** Clinical characteristics of atopic patients (AD) and control subjects (non AD).

AD						Non-AD			
AD subjects	Age (months)	SCORAD	Total IgE (kUI/l)	anti Der p1 IgE (kU/L)	Sampled inflammatory area	Control subjects	Age (months)	Total IgE (kUI/l)	anti Der p1 IgE (kU/L)
P01	25.9	26.7	76	<0.10	hand	P05	33.7	7	<0.10
P02	34.4	31.6	6392	>100.00	face	P08	16.2	ND	ND
P03	16.9	21.5	ND	ND	Back of thigh	P13	24.3	35	<0.10
P04	35.9	20.7	3413	>100.00	popliteal fossa	P14	36.0	11	<0.10
P06	33.4	25.6	128	1.41	antecubital fossa	P17	21.9	472	<0.10
P07	22.8	25.5	1055	>100.00	face	P18	24.7	67	<0.10
P09	16.7	22.2	39	5.53	antecubital fossa	P21	18.0	117	<0.10
P10	36.3	25.5	308	>100.00	torso	P22	31.2	5	<0.10
P11	22.4	22.1	1564	0.22	antecubital fossa	P23	15.2	8	<0.10
P12	13.7	25	7	<0.10	thigh	P24	17.3	2	<0.10
P15	35.5	33.7	3490	>100.00	antecubital fossa	P25	16.4	5	<0.10
P16	16.1	27.2	ND	7.59	thigh	P27	31.6	15	<0.10
P19	24.0	31	855	0.20	antecubital fossa	P28	35.9	<2	<0.10
P20	35.6	23.5	36	<0.10	antecubital fossa	P31	34.1	6	<0.10
P26	23.5	37.2	48	1.73	torso	P32	28.4	7	<0.10
P29	19.5	20.2	8	<0.10	antecubital fossa	P34	36.5	52	<0.10
P30	30.5	24.5	67	<0.10	antecubital fossa	P37	18.6	9	<0.10
P35	12.1	24.5	1352	0.12	back				
P36	23.2	27.4	200	9.59	antecubital fossa				
P38	15.8	22	30	13.10	antecubital fossa				
P39	12.4	31.2	1305	1.13	torso				

Results from transcript profiling of the most representative genes involved in barrier integrity, inflammation and host response are shown in S1 Table (shows fold change (FC) in AD *vs* non-AD subjects). Although the skin scratching procedure restricted our analysis to the upper epidermal layers, we found a distinctive inflammatory profile of AD patients, as previously demonstrated in [17]. Expression of the epidermal differentiation complex (EDC) genes *filaggrin* and *loricrin* was reduced in AD (FC~33 and ~38 respectively) contrary to the *S100A7* (FC~6), *A8* (FC~28) and *A9* (FC~21) genes that are known to take part in antimicrobial responses and the chemotaxis of T cells and neutrophils. Genes of the NLRP3 inflammasome, including *NLRP3* (FC~280) and its downstream products *Caspase-1* (FC~7) and *IL-1β* (FC~11), were also modulated, possibly in response to *S*. *aureus* infection and endogenous danger signals from tissue injury [18]. Expression of the antimicrobial peptides LL37 (FC~120), HBD2 (FC~7) and HBD3 (FC~12) was also upregulated, a feature usually identified as a response of keratinocytes to inflammation and bacterial infection [19]. Conversely, the down-regulation of *RNase7* expression (FC~6) agrees with data demonstrating that high levels of expression of *RNase7* in healthy skin contributes to the killing of *S*. *aureus* [20]. An increased expression of the chemokines CCL17 (FC~10), CCL3 (FC~12) and IL-8 (FC~32), and cytokines IL-13 (FC~643) and IL-22 (FC~691), suggests an active recruitment of immune cells such as monocytes, neutrophils, and lymphocytes of the Th2 and Th22 phenotypes.

**Fig 2:**
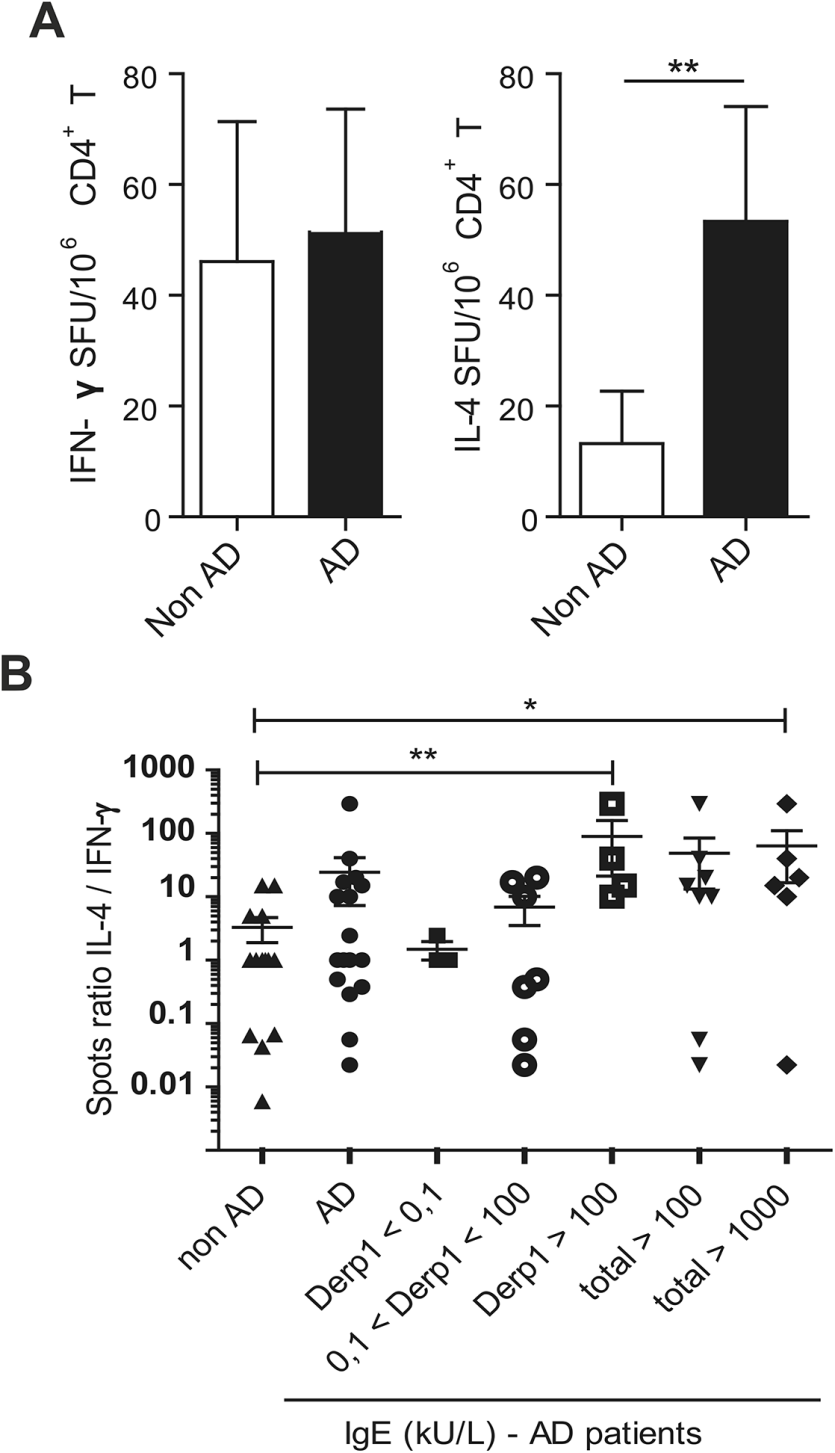
IL-4-producing peripheral T CD4^+^ cells against Der p allergens are increased in AD children compared to IFN-γ- producing T CD4^+^ cells. (A) IFN-γ and IL-4 ELISPot assays (spot forming units/10^6^ T cells) were performed on peripheral blood from non-AD (N = 14) and AD (N = 15) children in response to crude extracts of Der p. (B) Ratio of IL-4 *vs* IFN-γ CD4^+^ T cell spots relative to titers of total or Der p1 specific IgE antibodies (kU/ml) in AD patients. Mann-Whitney, mean values with SEM are shown, p*<0.05, p**<0.01.

These transcript analyses results are suggestive of a high rate of Th2 lymphocytes presence in the inflamed skin of AD children. Since T cells can continuously recirculate between the blood and inflamed lesions, and since we did not extract cells from the skin of children due to their age, we determined the phenotype of peripheral anti-Der p CD4^+^ T cells by IL-4 and IFN-γ ELISpots. Due to the small volumes of the available blood samples we were not able to analyze IL-22-producing CD4^+^ T cells. However, whereas CD4^+^ T cells co-cultured with autologous moDC pulsed with Der p allergens secreted IFN-γ in both AD and non-AD children, the number of IL-4-secreting-forming units was significantly higher in AD than non-AD children (Fig 2A). A prevalence of IL-4-secreting cells was observed in patients with Der p1-specific IgE>100 kUI/ml (Fig 2B) and this correlated with *S*. *aureus* counts (S1 Fig, Spearman’s correlation r = 0.63, p = 0.0062). As a whole, our analyses of the cutaneous microbiota, of transcript profiling and of the peripheral anti-Der p CD4^+^ T cell response in AD children have revealed a spectrum of clinical phenotypes including the colonization of inflammatory skin with *S*. *aureus*, defects in barrier integrity, inflammasome activation and leukocyte activation characterized by Th2/Th22 cells.

### The secretomes of cutaneous *S*. *aureus* and *S*. *epidermidis* exert opposite effects on moDC

**Fig 3:**
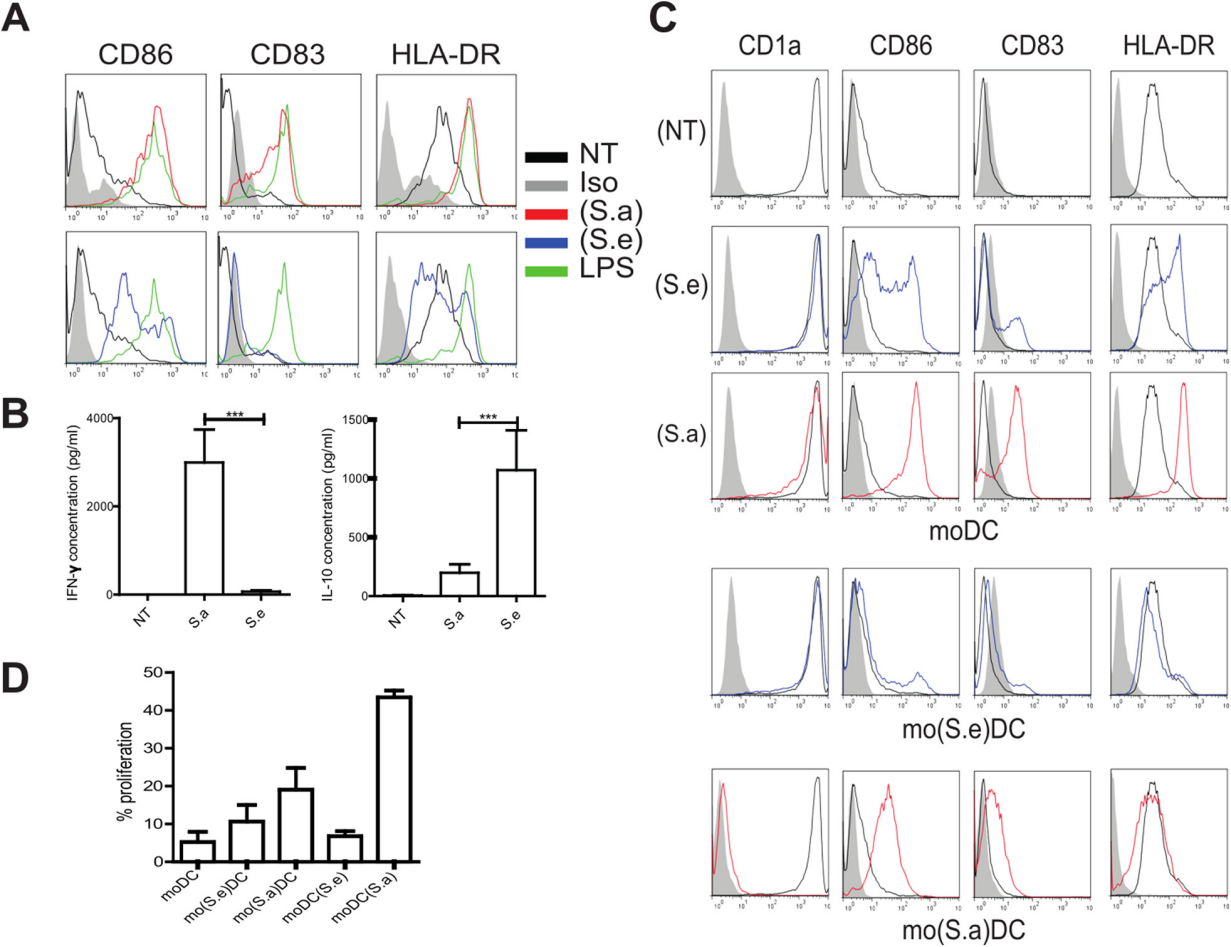
Factors secreted by cutaneous *S*. *aureus* and *S*. *epidermidis* exert opposite effects on moDC. (A) Representative activation phenotype (CD86, CD83 and HLA-DR levels) of monocyte-derived dendritic cells (moDC) exposed for 24 hours to the *S*. *aureus* (S.a, red) or *S*. *epidermidis* (S.e, blue) secretomes obtained from subject P04, or medium (NT, black) or LPS (green). (B) Secreted IFN-γ and IL-10 (pg/ml) levels in moDC as described in (A). Wilcoxon signed-rank test, mean values with SEM are shown, p*** <0.001, N = 14 independent experiments. (C) (S.e) and (S.a) were added to either moDC [moDC(S.e) and moDC(Sa)] or monocytes on day 0 of differentiation (mo(S.e)DC and mo(S.a)DC). (D) Cells were co-cultured with CFSE-labeled allogeneic naïve CD4^+^ T cells for 5 days at a ratio of 1:10 stimulator to T cells. The percentage of proliferating cells was then determined by flow cytometry. Mean values with +/- SEM are shown, N = 4, one-way ANOVA test p = 0.0009.

We next addressed whether *S*. *aureus* could influence the CD4^+^ T cell response by interfering with the activation of DC and their differentiation from monocytes. The recruitment of monocytes and their differentiation into DC in inflamed skin are crucial in the protection against pathogens. MoDC are particularly competent at activating skin-tropic T cells and thereby contribute to immune response and tolerance [21]. Secretome samples of *S*. *aureus* (S.a) were obtained by conditioning medium with *S*. *aureus* clones isolated from the skin of AD subject P04 who was highly colonized with *S*. *aureus* and showed a low bacterial diversity. To address the hypothesis that an imbalance in pathogen *versus* commensal bacteria is a determining factor that may affect moDC function, we also obtained *S*. *epidermidis* secretome samples (S.e) from the same subject. MoDC treated with (S.a) exhibited a CD86^high^CD83^high^HLA-DR^high^ mature phenotype (Fig 3A) similar to that observed upon stimulation with lipopolysaccharide (LPS). In contrast, cells stimulated with (S.e) expressed lower levels of CD86, CD83 and HLA-DR compared to either cells exposed to (S.a) or LPS or untreated cells which expressed HLA-DR only. Similar phenotypes were observed with (S.a) and (S.e) obtained from bacteria isolated from other children (*S*. *aureus* was also obtained from P02, P35, P36 and P39 and *S*. *epidermidis* from P02, P18, P23, P39, data not shown). Both (S.a) and (S.e) induced IL-6 and TNF-α secretion (not shown). (S.a) also induced the secretion of IFN-γ and low levels of IL-10, whereas (S.e) induced IL-10 but not IFN-γ secretion (Fig 3B *)*. An increase in HLA-DR expression and IFN-γ production by moDC was also observed when cells were pulsed with a mixture of recombinant enterotoxins that had been identified in the *S*. *aureus* secretome of patient P04 (S2 Fig). Further quantification of the toxins from the secretome of patient P04 (Fig 1C) showed that the *egc* gene products were present in amounts below 2.0 ng/ml while the SEC toxin was present at approximately 500 ng/ml. These data suggest that enterotoxins secreted by *S*. *aureus* take part in the process leading to IFN-γ secretion. In addition, moDC purified by flow cytometry that were stimulated with either (S.a) or (S.e) released IL-6 in both cases but differed in their ability to secrete IFN-γ and IL-10 (S3 Fig), providing evidence of the unusual capacity of moDC to secrete IFN-γ, as previously demonstrated [22].

The addition of (S.a) to monocytes before their differentiation resulted in CD1a^neg^CD83^low^CD86^high^HLA-DR^low^ moDC (mo(Sa)DC), a phenotype corresponding to semi-mature cells which contrasted with the fully mature phenotype observed when differentiated moDC were treated with (S.a) (moDC(Sa)) (Fig 3C). Monocytes treated with (S.e) differentiated into CD1a^pos^CD83^neg^CD86^neg^HLA-DR^low^ moDC (mo(Se)DC). To address the outcomes of these modifications on the ability of moDC to induce the proliferation of T lymphocytes, cells were co-cultured with allogeneic CFSE-labeled CD4^+^ T cells. When compared to untreated and (S.e)-treated moDC, cells incubated with (S.a) showed a huge increase in the proliferation of T cells, most likely because of a superantigenic effect. MoDC derived from (S.a)-treated monocytes were also less efficient than (S.a)-treated moDC in stimulating the proliferation of T cells (Fig 3D). Overall these observations are in agreement with respective moDC phenotype. Therefore we addressed the effects of the simultaneous presence of the *S*. *aureus* and *S*. *epidermidis* secretomes on the activation of moDC and their functional impact.

### MoDC stimulated with the *S*. *epidermidis* secretome releases IL-10 that impairs their maturation and reduces the effect of the *S*. *aureus* secretome

**Fig 4:**
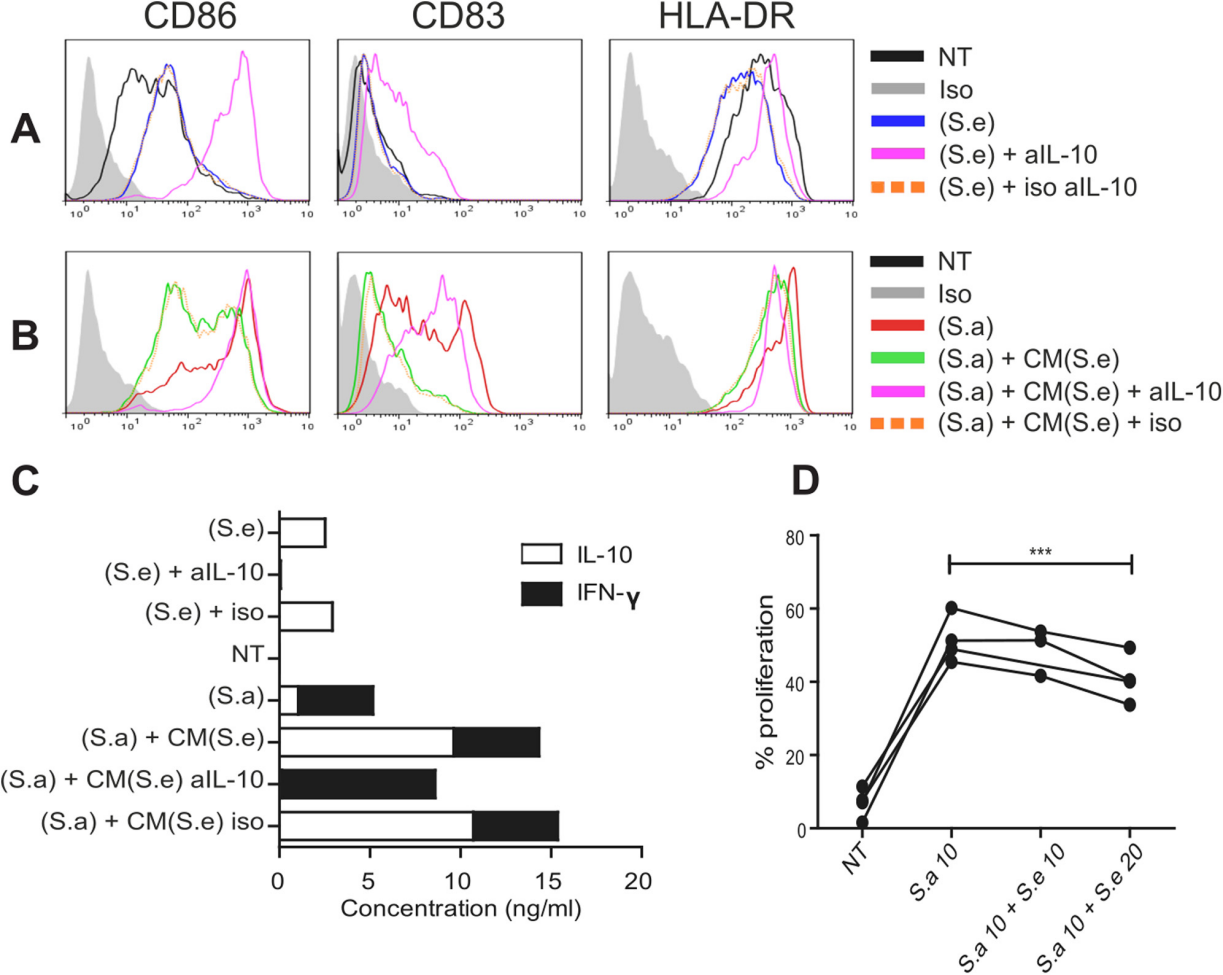
*S*. *epidermidis* stimulated moDC release IL-10 that impairs their maturation and reduces *S*. *aureus effect*. (A) Activation profile (CD86, CD83 and HLA-DR levels) of moDC exposed to either medium alone (NT), or the secretomes of *S*.*aureus* (S.a) and *S*.*epidermidis* (S.e) supplemented with anti-IL-10 or isotype-matched control (iso) antibodies. (B) MoDC were incubated with (S.a) alone or supplemented with conditioned medium (CM) from allogeneic moDC pulsed with (S.e), with the addition of anti-IL-10 or isotype-matched control antibodies. (C) Levels of secreted IFN-γ and IL-10 (ng/ml) by the same moDC presented in (A) and (B). Similar results were obtained in 3 independent experiments. (D) MoDC were exposed to a mixture of (S.a) at m.o.i of 10 (S.a 10) and increasing amounts of (S.e) co-cultured with CFSE-labeled allogeneic CD4^+^ T cells. The proliferation of T cells was quantified by flow cytometry (%). Paired t-test, p***<0.001 N = 4.

Stimulation of moDC with (S.e) in the presence of anti-IL-10 antibodies increased the expression of CD86, CD83 and HLA-DR (Fig 4A), demonstrating that IL-10 exerts a strong anti-inflammatory effect. In contrast, the addition of conditioned medium obtained from (S.e)-treated moDC to allogeneic (S.a)-treated moDC down-regulated the expression of HLA-DR, CD86 and CD83 (Fig 4B), an observation consistent with the higher amounts of IL-10 found in the medium. Moreover, the phenotypic changes shown in Fig 4A and 4B correlated with the relative amounts of IFN-γ and IL-10 quantified in the matched cell culture medium (Fig 4C). To assess the functional consequences of the simultaneous addition of the *S*. *aureus* and *S*. *epidermidis* secretomes on T cell proliferation, moDC were pulsed with mixtures of (S.e) and (S.a) at various ratios and then co-cultured with CFSE-labeled allogeneic CD4^+^ T cells. The addition of increasing amounts of (S.e) to a given quantity of (S.a) significantly decreased T cell proliferation (Fig 4D), suggesting that commensals such as *S*. *epidermidis* may play a prominent role in controlling the inflammatory effects of cutaneous *S*. *aureus*.

### The exposure of Treg cells to the *S*. *aureus* secretome impairs their suppressive activity

**Fig 5:**
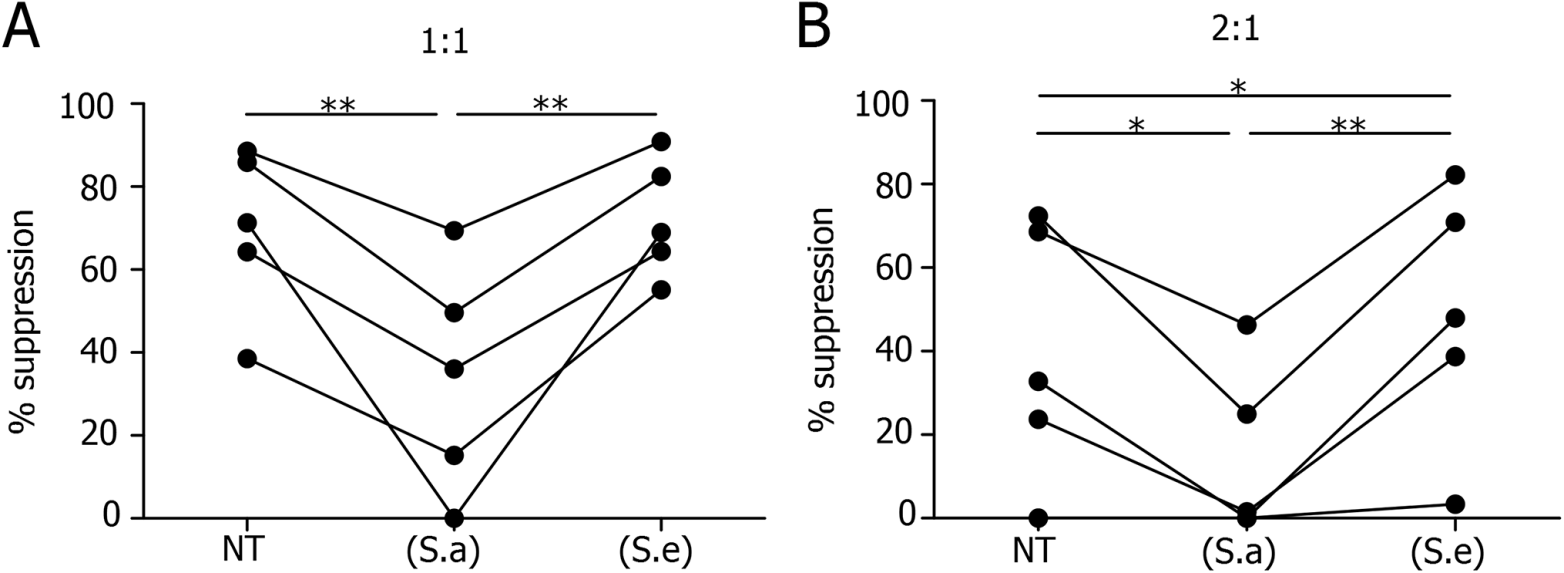
The *S*. *aureus* and *S*. *epidermidis* secretomes exert opposite effects on the Treg suppressive function. CFSE-labeled conventional CD4^+^ T cells (Tconv) were cultured in the presence of beads pre-loaded with anti-CD2, -CD3 and -CD28 antibodies and CD4^+^CD25^+^CD127^dim/-^ T cells (Treg cells expressing Foxp3) pretreated for 24 hours with medium only (NT), or the secretomes of *S*. *aureus* (S.a) or *S*. *epidermidis* (S.e) at Tconv to Treg ratios of 1:1 (A) and 2:1 (B). Proliferation was assessed by flow cytometry after 5 days and the percentage of suppression was calculated as (1—proliferation of Tconv with Treg / proliferation of Tconv without Treg) X100. Paired t-test, p*<0.05, p**<0.01, N = 5.

Treg cells play a key role in controlling skin inflammation therefore we investigated whether the *S*. *aureus* secretome could interfere with their function. To address this question Foxp3^+^CD4^+^CD25^+^CD127^dim/neg^ Treg cells were exposed to (S.a) or (S.e) for 24 hours before mixing with CFSE-labelled conventional CD4^+^ T cells (Tconv). Fig 5 shows that (S.a) dramatically reduced the suppressive activity of Treg cells at Tconv to Treg ratios of 1:1 and 2:1. In contrast, (S.e) increased Treg cell activity compared to untreated cells, which was observed at the 2:1 cell ratio. At a ratio of 1:1 the suppression activity of Treg cells was already very high and could not be improved by (S.e). Overall these data show that the secretome of *S*. *aureus* has a direct inhibitory effect on Treg cells, contrary to that of *S*. *epidermidis*.

## Discussion

Recent studies have demonstrated that the development and pathophysiology of AD is based upon interactions between cutaneous microbiota and skin-associated immunity [6]. Metagenomic analyses of the microbiome of healthy and atopic skin have revealed that a dominance of the pathogenic bacteria *S*. *aureus* is a major contributor to the pathology of AD [13]. In contrast, commensals such as *S*. *epidermidis* could provide a protective skin signature through interactions with resident DC [23]. One of the goals of our study was to provide an overall picture of very young atopic children in terms of the composition of their skin microbiota and the characterization of the associated inflammation and immune response markers. Culturomic analysis confirmed the presence of *S*. *aureus* exclusively in AD patients, a unique feature associated with high levels of blood IgE. However, the majority of patients (62%) were not colonized by *S*. *aureus* and had low levels of IgE. We can postulate that these *S*. *aureus*-negative children were in a post-flare state of the disease at the time of skin sampling, as previously described [13] or that they differed from *S*. *aureus*-positive ones in the severity of the sampled inflamed area. Indeed, the *S*. *aureus*-positive and -negative groups of patients did not differ significantly in their SCORAD, an index providing an overall clinical evaluation of the disease. When investigating skin bacterial diversity no significant differences were observed between the two groups. A metagenomic analysis of inflamed, xerotic and normal areas is now under investigation to complement this culturomic approach and will be the scope of a future report. The present study confirms previous work showing the modulation of some of the best known markers for barrier damage, inflammation and activated Th2/Th22 [24]. In addition, we confirmed the prevalence of IL-4-secreting T cells of Th2 phenotype in AD patients [25]. The absence of IL-12 secretion by moDC (not shown) following stimulation with LPS, is in line with our previous observation made on monocytes [26]. Altogether our data confirm previous observations made by us and others regarding the prevalence of cutaneous *S*. *aureus* in AD and the role of superantigens in proliferation and commitment of Th2 cells [27]. Indeed, we have shown that superantigens play an important role in increasing MHC-II expression in moDC and thus exacerbating the proliferation of T cells. We can also consider that MHC-II could be induced on cells such as keratinocytes that may also contribute to amplify the proliferation of CD4+ T cells [25]. Exotoxins secreted by *S*. *aureus* take part in mechanisms of escape from the immune system to ensure survival and spreading within tissues [28] and the δ-toxin secreted by *S*. *aureus* has been shown to promote IgE and IL-4 production as well as inflammatory skin disease in a mouse model [29]. We therefore considered that the secretome of *S*. *aureus* could play a crucial role in the pathophysiology of AD and that monocytes and DC could be primary targets of these bacterial products due to their role in monitoring inflamed skin. Accordingly, we demonstrated *in vitro* that the *S*. *aureus* secretome can commit monocytes to differentiate into semi-mature CD1a^neg^ DC. Based on previous observations [30] it is possible that these cells have lost their ability to ingest bacteria, thus allowing *S*. *aureus* to escape the host response. Interestingly, it has been shown that interactions of resident Th2 cells with monocytes could drive their differentiation into Th2-promoting DC, creating an amplification loop that could worsen the disease [31]. To mimic the effects of cutaneous commensal and pathogenic bacteria on skin CD4^+^ T cells, we exposed moDC to the *S*. *aureus* and *S*. *epidermidis* secretomes. We found that *S*. *aureus* promoted IFN-γ secretion by moDC but we can assume that IFN-γ-producing Th1 cells could also take part in this amplification process since they have been identified in AD skin albeit at lower levels than Th2/Th22 [24]. In contrast, IL-10 secretion by DC exposed to *S*. *epidermidis* reflects the mutualism capacity of skin commensals to counteract the harmful effects of pathogens by decreasing the production of inflammatory cytokines and the expression of both co-stimulatory molecules and of MHC-II-superantigen complexes. Besides these commensal-mediated anti-inflammatory mechanisms, specific subsets of skin Treg cells could control the proliferation of inflammatory T cells [8, 32], including cytotoxic CD8^+^ T cells that have been proposed to induce keratinocyte apoptosis [33]. However, in psoriasis lesions these cells were found to be able to proliferate but were defective in their capacity to control inflammation [32]. In AD skin similar defects could take place in a cell contact-dependent pathway through the action of *S*. *aureus* toxins on monocytes [34]. In addition, the stimulation of TLR2 on Treg cells has been shown to reduce their function [35]. Even though Treg cells isolated from peripheral blood may not accurately represent resident skin T cells they should provide an indication of the effects seen on resident skin Treg cells since they also express TLR. The functional and molecular characterization of the mechanisms underlying the inhibitory effects of the *S*. *aureus* secretome on suppressing the activity of Treg cells is now under investigation in our laboratory. Moreover, the ability of the *S*. *epidermidis* secretome to enhance Treg activity and potentially overcome the effects of *S*. *aureus* also requires further exploration. Overall, our observations highlight the importance of IL-10 secretion by DC which could balance the silencing of the anti-inflammatory effects of resident Treg cells in the inflamed skin of AD patients (S4 Fig). Our findings support the use of topical therapeutic approaches against AD in supplying suitable conditions to favor the anti-inflammatory properties of skin commensals such as *S*. *epidermidis*, as exemplified in [36].

## Supporting Information

S1 Fig IFN-γ and IL-4 ELISPot assays. (PDF)

S2 Fig Activation profile of moDC exposed to a mixture of recombinant *S*. *aureus* toxins. (PDF)

S3 Fig Production of cytokines by highly purified moDC. (PDF)

S4 Fig Schematic representation of hypothetical *S*. *aureus* and *S*. *epidermidis* secretome effects on activation of skin resident CD4^+^ T cells. (PDF)

S1 File Supplementary methods. (DOCX)

S2 File Supplementary references. (PDF)

S1 Table Transcriptome analysis of genes expressed at the inflamed areas of AD patients compared to their non-AD counterparts. (PDF)
