# Correction: Effects of the *Staphylococcus aureus* and *Staphylococcus epidermidis* Secretomes Isolated from the Skin Microbiota of Atopic Children on CD4^+^ T Cell Activation

**DOI:** 10.1371/journal.pone.0144323

**Published:** 2015-11-30

**Authors:** Emeline Laborel-Préneron, Pascale Bianchi, Franck Boralevi, Philippe Lehours, Frédérique Fraysse, Fanny Morice-Picard, Motoyuki Sugai, Yusuke Sato'o, Cédric Badiou, Gérard Lina, Anne-Marie Schmitt, Daniel Redoulès, Christiane Casas, Christian Davrinche

## Notice of Republication

This article was republished on November 17, 2015, to replace incorrectly published Supporting Information files. The originally published, uncorrected Supporting Information files and the republished, corrected Supporting Information files are provided here for reference.

## Supporting Information

S1 Zip Originally published, uncorrected Supporting Information files. (ZIP)

S2 Zip Republished, corrected Supporting Information files. (ZIP)
