# Supplementary material for: Effects of the Staphylococcus aureus and Staphylococcus epidermidis Secretomes Isolated from the Skin Microbiota of Atopic Children on CD4+ T Cell Activation
Source: PLoS One. 2015 Oct 28;10(10):e0141067. doi: 10.1371/journal.pone.0141067 (PMC4624846; doi:10.1371/journal.pone.0141067)
Supplement: S1 Fig — (PDF) [file pone.0141067.s001.pdf]

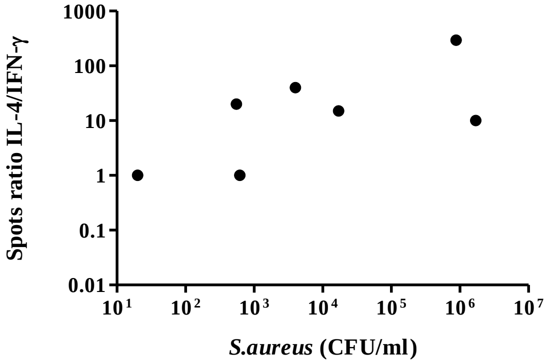

Figure S1. IFN-γ and IL-4 ELISPot assays. Spot forming units/10^6 T cells from the peripheral blood of AD and non-AD children in response to crude extract (CE) of Der p were quantified. In AD patients (N=17), counts (CFU/ml) of skin *S. aureus* were associated with the IL4 to IFN-γ spots ratio. Spearman's correlation, r=0.63, p=0.0062.
