# Supplementary material for: Effects of the Staphylococcus aureus and Staphylococcus epidermidis Secretomes Isolated from the Skin Microbiota of Atopic Children on CD4+ T Cell Activation
Source: PLoS One. 2015 Oct 28;10(10):e0141067. doi: 10.1371/journal.pone.0141067 (PMC4624846; doi:10.1371/journal.pone.0141067)
Supplement: S2 Fig — (PDF) [file pone.0141067.s002.pdf]

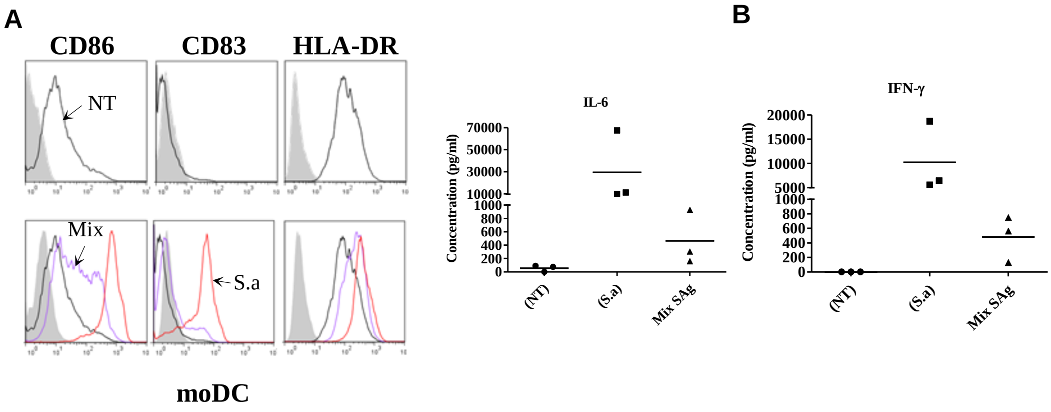

Figure S2. Activation profile of moDC exposed to a mixture of recombinant *S. aureus* toxins. (A) Activation phenotype (CD86, CD83 and HLA-DR levels) of moDC exposed to medium (NT), *S. aureus* secretome (S.a) or a mixture of recombinant *S. aureus* toxins SEC, SEG, SEI, SElM, SElN and SElO (Mix) at 100 ng/ml each for 24 hours. (B) Quantification (pg/ml) of IL-6 and IFN-γ secreted by cells in (A). N=3 independent experiments.
