# Supplementary material for: Effects of the Staphylococcus aureus and Staphylococcus epidermidis Secretomes Isolated from the Skin Microbiota of Atopic Children on CD4+ T Cell Activation
Source: PLoS One. 2015 Oct 28;10(10):e0141067. doi: 10.1371/journal.pone.0141067 (PMC4624846; doi:10.1371/journal.pone.0141067)
Supplement: S3 Fig — (PDF) [file pone.0141067.s003.pdf]

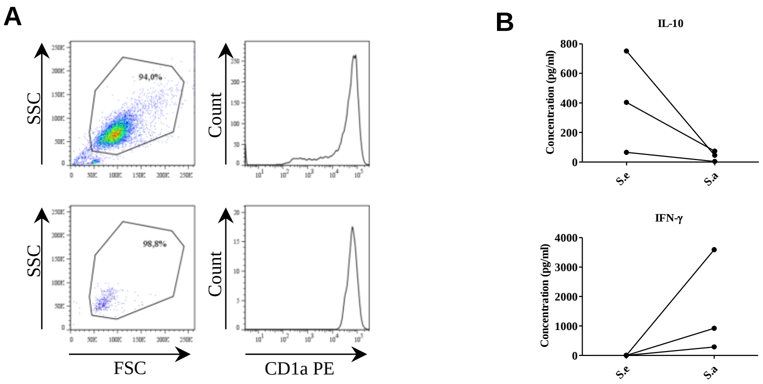

Figure S3. Production of cytokines by highly purified moDC. MoDC were sorted on the basis of CD1a expression (A) and exposed to *S.aureus* (S.a) and S. *epidermidis* (S.e) secretomes for 24 hours. IFN-γ and IL-10 secreted by purified cells were quantified (pg/ml) by a cytometry bead assay (B). The experiment shown in (A) is representative of three independent ones.
