# Supplementary material for: Effects of the Staphylococcus aureus and Staphylococcus epidermidis Secretomes Isolated from the Skin Microbiota of Atopic Children on CD4+ T Cell Activation
Source: PLoS One. 2015 Oct 28;10(10):e0141067. doi: 10.1371/journal.pone.0141067 (PMC4624846; doi:10.1371/journal.pone.0141067)
Supplement: S4 Fig — (PDF) [file pone.0141067.s004.pdf]

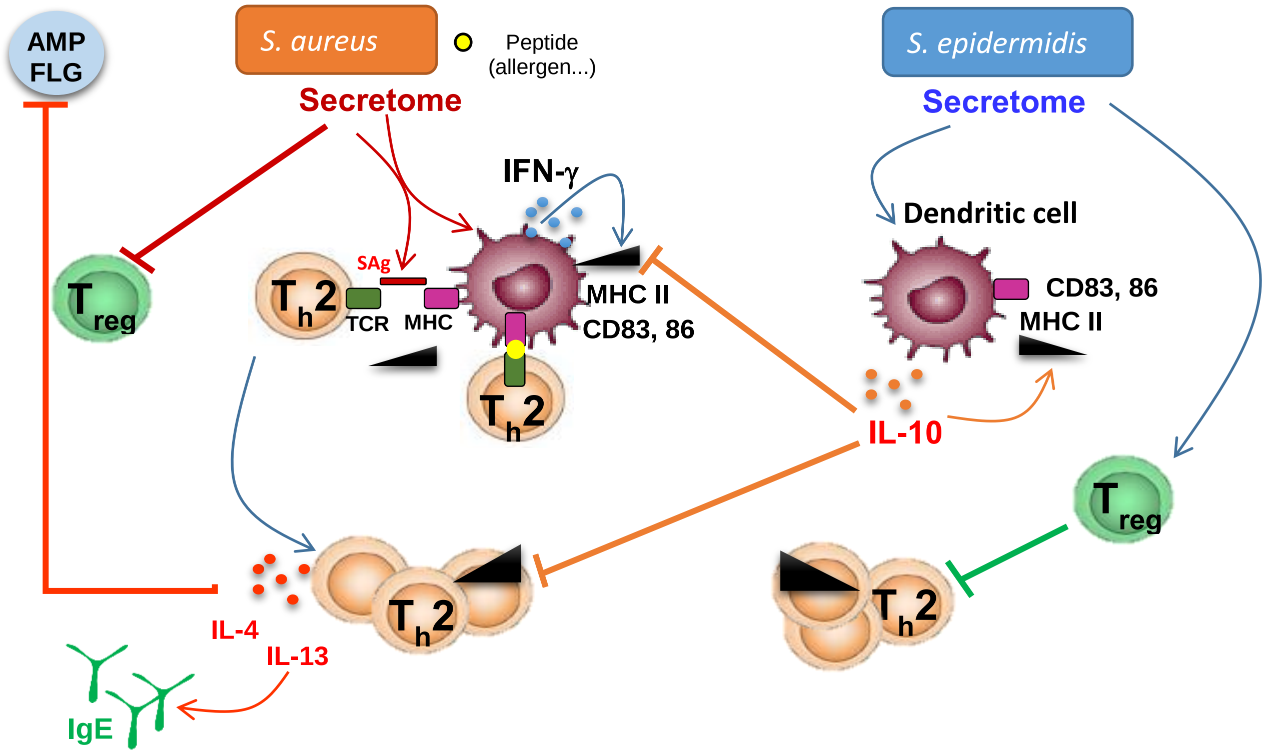

Figure S4. Schematic representation of hypothetical *S. aureus* and *S. epidermidis* secretome effects on activation of skin resident CD4$^+$ T cells. Exposure to the *S. aureus* secretome, which includes superantigen toxins (Sag), induces IFN-γ secretion by dendritic cells and increases the expression of MHC-II to allow the presentation of more antigens and SAg. This leads to the expansion of resident T cells, most of which are Th2 in AD. Th2-mediated secretion of IL-4 and IL-13 supports the production of IgE, barrier dysfunction (FLG) and inhibition of antimicrobial peptides supply (AMP). The harmful effects of the *S. aureus* secretome can be amplified by direct inhibition of regulatory T cell (Treg) activity. However, these deleterious effects can be counteracted by IL-10 secreted by dendritic cells which are exposed to skin commensals such as *S. epidermidis.*
