# Supplementary material for: Effects of the Staphylococcus aureus and Staphylococcus epidermidis Secretomes Isolated from the Skin Microbiota of Atopic Children on CD4+ T Cell Activation
Source: PLoS One. 2015 Oct 28;10(10):e0141067. doi: 10.1371/journal.pone.0141067 (PMC4624846; doi:10.1371/journal.pone.0141067)
Supplement: S1 File — (DOCX) [file pone.0141067.s005.docx]

**S1 File. Supporting Methods**

**Real Time RT-PCR analysis on skin scratching**

Skin samples were taken by scratching the skin surface (5 times back and forth) with a sterile micro-abrasive tool (Vitry, Paris, France). Cell aggregates were transferred from the tool to a microtube containing 500 μL of RNAprotect Cell Reagent (QIAGEN, Courtaboeuf, France) and placed at -80°C.

Total RNA was isolated using the RNeasy mini QIAcube kit (QIAGEN, Courtaboeuf, France) according to the manufacturer’s directions, including DNase I digestion. RNA was eluted in 30 μL of sterile, RNase-free water. Ten μL of RNA was reverse transcribed to single-stranded cDNA by the High Capacity cDNA kit (Life Technologies, Saint Aubin, France), using random hexamer primers, in a final volume of 20 μL according to the manufacturer’s instructions. Amplification of skin samples was performed as described in [1].

Results are expressed as fold change 2(-deltadeltaCt), where deltaCt corresponds to the Ct of target gene minus the Ct of endogenous control (RLPL0) and deltadeltaCt correspond to deltaCt of the sample minus the deltaCt of the calibrator (healthy children).

Primers and probes used were designed by Life Technologies: HBD3-Hs00218678_m1, HBD2-Hs00175474_m1, BCL3-Hs00180403_m1, LL37-Hs00189038_m1, CASP1-Hs00354836_m1, CCL17-Hs00171074_m1, CCL3-Hs00234142_m1, FLG-Hs00856927_g1, IL10-Hs00961622_m1, IL13-Hs00174379_m1, IL1B-Hs01555410_m1, IL22-Hs01574154_m1, IL8-Hs00174103_m1, LOR-Hs01894962_s1, NLRP3-Hs00918082_m1, Elafin-Hs00160066_m1, RNASE7-Hs00922963_s1, RPLP0-Hs99999902_m1, S100A7-Hs00161488_m1, S100A8-Hs00374263_m1, S100A9-Hs00610058_m1.

**Preparation and characterization of secretomes**

Secretomes were prepared with *S. aureus* and *S. epidermidis* clones isolated from children by overnight culture in RPMI 1640 medium supplemented with 10% FCS (inoculation at OD600nm=0.05) until stationary phase was reached. Bacterial suspension was centrifuged, the supernatant was filtered (0.22μm) and 1% penicillin-streptomycin was added. Final numbers of bacteria (CFU/ml) were assessed for each culture. Expression of recombinant staphylococcal enterotoxins (SE) was carried out with pKNX, pKOX and pKC2X1 [2, 3]. Preparation of antibody for ELISA and Sandwich ELISA was carried out as described [4] with some modifications. Briefly, StartingBlock (PBS) Blocking Buffer (Thermo) for blocking, Can Get Signal® Immunoreaction Enhancer Solution (TOYOBO) for dilutions of antibody and samples, SuperSignal ELISA Femto Maximum Sensitivity Substrate (Thermo) as substrate, Varioskan™ Flash Multimode Reader (Thermo) for reading luminescence were used. The calibration curve was prepared with 0.2-20.0 ng/ml recombinant protein. The concentration of SE was determined by converting the capsular polysaccharide (CPS) to the corresponding concentrations by using the standard curve.

**Identification of enterotoxins genes and** **production of recombinant proteins**

*DNA microarray assay*.

Bacterial DNA was extracted according to the manufacturer’s recommended protocol using commercial extraction kits (Qiagen). The diagnostic DNA microarrays, *S. aureus* Genotyping kit (Alere), Identibac *S. aureus* Genotyping ® (Alere) was used for supernatigen gene detection, using procedures and protocols, as previously described in details [5]. For data interpretation, alleles of a same gene were pooled as one genotypic marker to avoid redundancy.

*Production of recombinant enterotoxins*

SEB, SEC, SEG, SEI, SElM, SElN and SElO were produced in Escherichia coli M15 as His-tagged recombinant toxins and purified by affinity chromatography on a nickel affinity column according to the supplier's instructions (New England Biolabs, Ipswich, USA) as previously described [6]. Protein purity was verified by SDS-PAGE. LPS was removed from toxin solutions by affinity chromatography (Detoxi-GEL endotoxin Gel®, Pierce Rockford, USA). The QCL-1000 Limulus amebocyte lysate assay® (Cambrex-BioWhittaker, Walkersville, USA) showed that the endotoxin content of the recombinant SE solutions was less than 0.005 units/ml. Superantigenic activities of toxins were assessed by measuring CD69 surface expression by T cells.

**Generation and culture of cells**

***Monocytes and monocyte-derived dendritic cells (moDC)***

Peripheral blood mononuclear cells (PBMC) were isolated from buffy coats (blood donors from EFS, Toulouse) or from patients/controls blood by Ficoll separation (PAA). Monocytes were isolated from PBMC with the use of CD14 microbeads and a magnetic bead separation system (Miltenyi). Monocytes were maintained for 5 days in RPMI medium supplemented with 10% FCS, 1% penicillin-streptomycin, 20 ng/ml recombinant GM-CSF (Miltenyi) and 50 ng/ml IL-13 (donated by Sanofi-Aventis). On day 5, non-adherent monocytes-derived dendritic cells (moDC) were recovered and stimulated for 24 hours with either *S. aureus* and *S. epidermidis* secretome at a multiplicity of infection (MOI) of 20 or 5% (v/v), unless otherwise stated, or purified toxins at 100 ng/ml, LPS at 100 ng/ml, with or without anti-IL-10 antibody or matched isotype (eBioscience, 16-7108 and 16-4301) at 10 μg/ml. In experiments with highly purified cells, moDC were generated as described, stained on day 5 with anti CD1a PE antibody and sorted by flow cytometry (Cell sorter FACS ARIA-SORP). Alternatively, monocytes were stimulated on day 0, supplemented with GM-CSF and IL-13, maintained for 5 days in differentiation medium and then treated/used as described above for moDC. Cells were stained and submitted to flow cytometry analysis on a FACSCalibur cytometer (BD Biosciences) as detailed in the Flow cytometry analysis section.

**T cell ELISpot assays**

For ELISpot assays, monocytes and autologous CD4^+^ T cells were isolated from patients/controls blood within 24h after sampling. CD4^+^ T cells were kept at -80°C in FCS 10% DMSO over the course of moDC generation. On day 5, moDC were harvested, washed and stimulated with either crude extracts of *D. pteronyssinus* (Der p) at 500 ng/ml (LTN-DPE-4, Indoor Biotechnologies), or recombinant allergen Der p1 at 100 ng/ml (RP-DP1D-1, Indoor Biotechnologies). Alternatively purified *S. aureus* enterotoxin SEB was used at 100 ng/ml. T cells and autologous moDC were co-cultured for 20 hours in ELISpot plates (Diaclone) coated with anti-IFN-γ or -IL-4 capture antibodies. After cell removal by washing, biotinylated detection antibodies, enzyme-conjugated streptavidin and substrate are successively added according to manufacturer’s instructions. Plates were read using the C.T.L. analyzer and the Immunospot software. Results were expressed as numbers of spots per 10^6^ T cells.

**Statistical Analysis**

Statistical analysis were performed using SAS® software, release 9.3. All quantitative variables were expressed as sample size, mean, standard deviation (SD), median and range values. Intergroup comparison of clinical and gene expression were performed with Student’s t-test, Mann-Whitney’s test or Wilcoxon’s test, depending on the normality of distributions as specified. Correlations between clinical parameters and qPCR-quantified genes with a differential expression fold change (FC) >2 and a correlation coefficient >│0.6│ (i.e. meaning a strong correlation according to JD Evans’ guide) were selected and analysed using Pearson’s or Spearman’s correlation test (r). All statistical tests were performed at a significance level of 0.05.
