# Supplementary material for: Effects of the Staphylococcus aureus and Staphylococcus epidermidis Secretomes Isolated from the Skin Microbiota of Atopic Children on CD4+ T Cell Activation
Source: PLoS One. 2015 Oct 28;10(10):e0141067. doi: 10.1371/journal.pone.0141067 (PMC4624846; doi:10.1371/journal.pone.0141067)
Supplement: S1 Table — (PDF) [file pone.0141067.s007.pdf]

S1 Table. Trancriptome analysis of gene expressed in inflamed areas of AD patients relative to their non-AD counterparts.

Gene	p value	FC
**FLG**	<.0001	-33,4620368
**LOR**	<.0001	-37,8871287
**S100A7**	0.0018	6,69256792
**S100A8**	0.0002	28,2174085
**S100A9**	0.0002	21,1013902
**NLRP3**	0.0003	280,416897
**CASP1**	0.0612	-6,59843344
**IL1β**	0.0412	10,8693377
**LL37**	0.0044	119,54566
**HBD2**	0.0037	6,6670778
**HBD3**	0.0029	11,8114083
**RNASE7**	0.0008	-6,07797538
**CCL3**	0.0306	12,5081269
**CCL17**	0.0181	10,2317651
**IL-8**	0.0009	32,1584301
**IL-13**	0.0009	643,855732
**IL-22**	0.0019	693,153442

Skin samples were taken by scratching the skin surface and reverse-transcribed RNA submitted to PCR with primers for genes as indicated. Results are expressed as Fold Change (FC) in AD (N=21) vs non-AD samples (N=14). Associated p values are shown according to methods described in statistical section**.**
