# Supplementary material for: Correction: Effects of the Staphylococcus aureus and Staphylococcus epidermidis Secretomes Isolated from the Skin Microbiota of Atopic Children on CD4+ T Cell Activation
Source: PLoS One. 2015 Nov 30;10(11):e0144323. doi: 10.1371/journal.pone.0144323 (PMC4664274; doi:10.1371/journal.pone.0144323)
Supplement: S1 Zip — (ZIP) [file pone.0144323.s001.zip › S1_Table.docx]

**S1 Table**. **Transcriptome analysis of genes expressed at the inflamed areas of AD patients compared to their non-AD counterparts**. Skin samples were obtained by scratching the skin surface. RNA was isolated and reverse-transcribed then analyzed using PCR with primers for the genes as indicated. Results are expressed as Fold Change (FC) in AD (N=21) vs non-AD samples (N=14). Associated p values are shown which were obtained as described in the Statistical Analysis section of the Materials and Methods.
