# Supplementary material for: Correction: Effects of the Staphylococcus aureus and Staphylococcus epidermidis Secretomes Isolated from the Skin Microbiota of Atopic Children on CD4+ T Cell Activation
Source: PLoS One. 2015 Nov 30;10(11):e0144323. doi: 10.1371/journal.pone.0144323 (PMC4664274; doi:10.1371/journal.pone.0144323)
Supplement: S1 Zip — (ZIP) [file pone.0144323.s001.zip › S2_File.docx]

S2 File. Supporting References

1. Bianchi P, Ribet V, Casas C, Lejeune O, Schmitt AM, Redoules D. Analysis of gene expression in atopic dermatitis using a microabrasive method. The Journal of investigative dermatology. 2012;132(2):469-72.

2. Omoe K, Hu DL, Ono HK, Shimizu S, Takahashi-Omoe H, Nakane A, et al. Emetic potentials of newly identified staphylococcal enterotoxin-like toxins. Infection and immunity. 2013;81(10):3627-31.

3. Hu DL, Omoe K, Shimoda Y, Nakane A, Shinagawa K. Induction of emetic response to staphylococcal enterotoxins in the house musk shrew (Suncus murinus). Infection and immunity. 2003;71(1):567-70.

4. Omoe K, Ishikawa M, Shimoda Y, Hu DL, Ueda S, Shinagawa K. Detection of seg, seh, and sei genes in Staphylococcus aureus isolates and determination of the enterotoxin productivities of S. aureus isolates Harboring seg, seh, or sei genes. Journal of clinical microbiology. 2002;40(3):857-62.

5. Monecke S, Luedicke C, Slickers P, Ehricht R. Molecular epidemiology of Staphylococcus aureus in asymptomatic carriers. European journal of clinical microbiology & infectious diseases : official publication of the European Society of Clinical Microbiology. 2009;28(9):1159-65.

6. Thomas D, Dauwalder O, Brun V, Badiou C, Ferry T, Etienne J, et al. Staphylococcus aureus superantigens elicit redundant and extensive human Vbeta patterns. Infection and immunity. 2009;77(5):2043-50.
