# Supplementary material for: Correction: Effects of the Staphylococcus aureus and Staphylococcus epidermidis Secretomes Isolated from the Skin Microbiota of Atopic Children on CD4+ T Cell Activation
Source: PLoS One. 2015 Nov 30;10(11):e0144323. doi: 10.1371/journal.pone.0144323 (PMC4664274; doi:10.1371/journal.pone.0144323)
Supplement: S1 Zip — (ZIP) [file pone.0144323.s001.zip › S4_Fig.docx]

**S4 Fig . Schematic representation of hypothetical S. aureus and S. epidermidis secretome effects on activation of skin resident CD4+ T cells.**

Exposure to the S. aureus secretome, which includes superantigen toxins (SAg), induces IFN-γ secretion by dendritic cells and increases the expression of MHC-II to allow the presentation of more antigens and SAg. This leads to the expansion of resident T cells, most of which are Th2 in AD. Th2-mediated secretion of IL-4 and IL-13 supports the production of IgE, barrier dysfunction (FLG) and inhibition of antimicrobial peptides supply (AMP). The harmful effects of the S. aureus secretome can be amplified by direct inhibition of regulatory T cell (Treg) activity. However, these deleterious effects can be counteracted by IL-10 secreted by dendritic cells which are exposed to skin commensals such as S. epidermidis.
