# Supplementary material for: Correction: Effects of the Staphylococcus aureus and Staphylococcus epidermidis Secretomes Isolated from the Skin Microbiota of Atopic Children on CD4+ T Cell Activation
Source: PLoS One. 2015 Nov 30;10(11):e0144323. doi: 10.1371/journal.pone.0144323 (PMC4664274; doi:10.1371/journal.pone.0144323)
Supplement: S1 Zip — (ZIP) [file pone.0144323.s001.zip › S3_Fig.docx]

**S3 Fig . Production of cytokines by highly purified moDC.**

MoDC were sorted on the basis of CD1a expression (A) and exposed to S.aureus (S.a) and S. epidermidis (S.e) secretomes for 24 hours. IFN-γ and IL-10 secreted by purified cells were quantified (pg/ml) by a cytometry bead assay (B). The experiment shown in (A) is representative of three independent ones.
