# Supplementary material for: Correction: Effects of the Staphylococcus aureus and Staphylococcus epidermidis Secretomes Isolated from the Skin Microbiota of Atopic Children on CD4+ T Cell Activation
Source: PLoS One. 2015 Nov 30;10(11):e0144323. doi: 10.1371/journal.pone.0144323 (PMC4664274; doi:10.1371/journal.pone.0144323)
Supplement: S1 Zip — (ZIP) [file pone.0144323.s001.zip › S1_Fig.docx]

**S1 Fig. IFN-γ and IL-4 ELISPot assays.**

Spot forming units/106 T cells from the peripheral blood of AD and non-AD children in response to crude extract (CE) of Der p were quantified. In AD patients (N=17), counts (CFU/ml) of skin S. aureus were associated with the IL4 to IFN-γ spots ratio. Spearman’s correlation, r=0.63, p=0.0062.
