# Supplementary material for: Correction: Effects of the Staphylococcus aureus and Staphylococcus epidermidis Secretomes Isolated from the Skin Microbiota of Atopic Children on CD4+ T Cell Activation
Source: PLoS One. 2015 Nov 30;10(11):e0144323. doi: 10.1371/journal.pone.0144323 (PMC4664274; doi:10.1371/journal.pone.0144323)
Supplement: S1 Zip — (ZIP) [file pone.0144323.s001.zip › S2_Fig.docx]

**S2 Fig. Activation profile of moDC exposed to a mixture of recombinant S. aureus toxins.**

(A) Activation phenotype (CD86, CD83 and HLA-DR levels) of moDC exposed to medium (NT), S. aureus secretome (S.a) or a mixture of recombinant S. aureus toxins SEC, SEG, SEI, SElM, SElN and SElO (Mix) at 100 ng/ml each for 24 hours. (B) Quantification (pg/ml) of IL-6 and IFN-γ secreted by cells in (A). N=3 independent experiments.
